# Sarcopenia in Chronic Heart Failure: Pathophysiology, Clinical Consequences, and Emerging Multimodal Therapeutic Strategies

**DOI:** 10.3390/nu18091431

**Published:** 2026-04-30

**Authors:** Dominik Kurczyński, Adam Załuczkowski, Helena Kalota, Brygida Przywara-Chowaniec, Andrzej Tomasik

**Affiliations:** 1 II Department of Cardiology, Faculty of Medical Sciences in Zabrze, Medical University of Silesia, Marii Skłodowskiej-Curie 10, 41-800 Zabrze, Poland; bprzywara-chowaniec@sum.edu.pl; 2 Doctoral School, Medical University of Silesia, Poniatowskiego 15, 40-055 Katowice, Poland; 3 School of Medicine, Medical University of Silesia, Poniatowskiego 15, 40-055 Katowice, Poland; s86350@365.sum.edu.pl (A.Z.); s84690@365.sum.edu.pl (H.K.)

**Keywords:** sarcopenia, heart failure, nutrition, muscle metabolism, protein intake, cachexia, exercise, amino acids

## Abstract

Sarcopenia is increasingly recognized as a key extracardiac manifestation of heart failure (HF), contributing to functional impairment, reduced quality of life, and adverse clinical outcomes. Characterized by progressive loss of skeletal muscle mass, strength, and physical performance, it affects more than half of hospitalized HF patients. It is independently associated with increased mortality and reduced exercise capacity. The pathophysiology of sarcopenia in HF is multifactorial and closely linked to metabolic and nutritional disturbances. Chronic inflammation, neurohormonal activation, oxidative stress, endothelial dysfunction, and anabolic resistance contribute to muscle catabolism and impaired protein synthesis. These alterations are further exacerbated by inadequate dietary protein intake and micronutrient deficiencies, promoting progressive muscle wasting and functional decline. Sarcopenia may also represent an early and potentially modifiable stage in the continuum toward cardiac cachexia. This narrative review provides a comprehensive synthesis of current evidence on the epidemiology, pathophysiological mechanisms, and management of sarcopenia in HF, with particular emphasis on nutritional and metabolic determinants. Emerging data support a multimodal therapeutic approach integrating exercise training with targeted nutritional strategies, including adequate protein intake, essential amino acid supplementation, and correction of micronutrient deficiencies. However, evidence from large, well-designed trials remains limited. In summary, improved recognition and integrated management of sarcopenia in HF are essential. Future research should focus on the development of effective, nutrition-centered therapeutic strategies.

## 1. Introduction

Heart failure (HF) is a complex clinical syndrome characterized by hallmark symptoms such as fatigue, reduced exercise tolerance, and exertional dyspnea and often accompanied by signs like peripheral congestion and elevated jugular venous pressure. These manifestations result from structural or functional cardiac abnormalities leading to reduced cardiac output and/or elevated intracardiac pressures at rest or during stress [1]. HF affects approximately 2% of the population in developed countries, with prevalence increasing markedly with age, doubling nearly every decade in men and every seven years in women beyond 55 years of age [2].

Historically, the conceptualization of HF has transitioned from a “hemodynamic” model, which focused primarily on the heart’s pumping capacity and peripheral vasoconstriction, to a “neurohormonal” model. This model recognizes that HF progression is driven by overactivation of neurohormonal pathways that exert deleterious effects on the heart and peripheral circulation in response to reduced cardiac output [3].

This paradigm shift has enabled the development and widespread use of pharmacological therapies such as angiotensin-converting enzyme (ACEI) inhibitors and beta-blockers, which have substantially improved survival and life expectancy in HF patients [1].

As a result of these therapeutic advances, an increasing number of individuals with HF survive into older age, facing new clinical challenges such as frailty and multi-morbidity, which were historically underrecognized in this population. Notably, HF is frequently accompanied by profound nutritional disorders and alterations in body composition, including sarcopenia—the age-related loss of skeletal muscle mass and strength—and cardiac cachexia, a wasting syndrome characterized by severe body weight, fat, and muscle loss.

These nutritional and muscular abnormalities significantly contribute to the poor prognosis observed in HF patients and highlight the need for comprehensive management approaches that extend beyond pharmacological treatment. Despite the recognition of these complex interrelations, standardized guidelines for the non-pharmacological management of HF, particularly those addressing nutritional status and muscle wasting, remain lacking. Understanding the pathophysiology linking HF with sarcopenia and cachexia is therefore critical for improving patient outcomes and developing integrated therapeutic strategies.

## 2. Literature Review Methodology

This narrative review provides a comprehensive overview of sarcopenia in heart failure, integrating evidence from clinical studies, meta-analyses, and international guidelines. Emphasis was placed on pathophysiological pathways and potential therapeutic strategies, particularly nutritional interventions. We searched PubMed and Google Scholar for articles published between 2010 and 2026 using combinations of the following terms: heart failure, sarcopenia, prevalence, mortality, pathophysiology, and treatment. Several articles were identified through the reference lists of key studies. Publications were selected based on their relevance and clinical significance.

## 3. Sarcopenia in Heart Failure: Prevalence and Mortality

The main wasting syndromes occurring in the course of chronic diseases, such as chronic heart failure (CHF), include sarcopenia and cachexia [4].

Sarcopenia is a disease of skeletal muscle characterized by reduced muscle strength, low muscle mass, or impaired muscle quality, leading to decreased physical performance [5]. Initially considered exclusively as a geriatric condition, it is now also associated with numerous chronic diseases. It is estimated that sarcopenia affects 10–16% of the elderly population worldwide, while its prevalence is higher among patients with chronic conditions. Depending on the clinical population studied, the prevalence of sarcopenia ranges from 18% in patients with diabetes to as high as 66% in individuals with unresectable esophageal cancer [6]. According to recent studies, signs of sarcopenia can be observed in 36% (10–64%) of patients with chronic heart failure (CHF), with a markedly higher prevalence among hospitalized patients (55%) compared with those treated in the outpatient setting (26%) [7].

Current data indicate a frequent coexistence of both conditions. Importantly, the presence of sarcopenia independently worsens prognosis in patients with CHF [8]. Von Haehling et al. demonstrated increased mortality among patients with CHF and concomitant sarcopenia compared with those without skeletal muscle abnormalities over 100 months of follow-up. At 24 months, mortality was 22.2% in patients with muscle wasting versus 10% in non-sarcopenic patients [9]. These findings are consistent with global statistics concerning patients with sarcopenia. Among individuals with muscle wasting, a significant increase in mortality (31%) was observed compared with non-sarcopenic patients (8%) over 55 months of follow-up. The number of hospitalizations was also higher in sarcopenic patients (68% vs. 48%). Moreover, an increased tendency toward injuries was noted in this population [10].

## 4. Diagnosing Sarcopenia in Heart Failure Patients

The diagnostic criteria for sarcopenia have evolved beyond the EWGSOP2 (2019) framework, which defines probable, confirmed, and severe sarcopenia based on muscle strength, muscle mass or quality, and physical performance [5]. More recent consensus statements have further refined this approach. In 2024, the Global Leadership Initiative on Sarcopenia (GLIS) proposed a conceptual definition in which muscle mass, muscle strength, and muscle-specific strength are considered core components of sarcopenia [11].

However, important limitations remain in the context of heart failure. Current diagnostic thresholds were derived mainly from community-dwelling populations and may not be fully applicable to HF patients, in whom fluid overload, reduced perfusion, and deconditioning affect muscle assessment [12]. In addition, no clearly defined preclinical stage of sarcopenia exists, which limits early identification.

In routine clinical practice, sarcopenia assessment relies mainly on functional and quantitative measures. Although imaging-based approaches such as CT or ultrasound can provide additional information on muscle structure and quality, they are not yet routinely implemented in HF care, and HF-specific reference values are lacking.

## 5. The Impact of Sarcopenia on Patients with CHF

Muscle loss in the course of heart failure leads to further deterioration of physical capacity beyond the symptoms of CHF. In patients with heart failure and sarcopenia, scores on the Short Physical Performance Battery (SPPB) and EQ-5D, as well as handgrip strength and quadriceps muscle strength, were lower in comparison with CHF patients without sarcopenia [13]. Additionally, reduced peak oxygen uptake (pVO_2_) is frequently observed. Contemporary studies indicate that lower pVO_2_ values, as well as the absence of improvement during treatment optimization, are associated with an increased risk of mortality and cardiovascular events [14,15]. Importantly, reduced peak oxygen uptake in patients with sarcopenia does not solely reflect impaired cardiac function but also peripheral skeletal muscle limitations. Reduced muscle mass, mitochondrial dysfunction, and impaired oxygen extraction contribute to a disproportionate reduction in exercise capacity [16,17].

Consequently, cardiopulmonary exercise testing (CPET) parameters—including pVO_2_, ventilatory efficiency (VE/VCO_2_ slope), and anaerobic threshold—reflect an interaction between central (cardiac) and peripheral (skeletal muscle) mechanisms [18]. This is clinically relevant, as these parameters are commonly used for risk stratification and to guide exercise prescription in heart failure.

These findings reinforce the concept that exercise limitation in heart failure reflects an interplay between central and peripheral mechanisms.

According to some authors, during the progression of HF, sarcopenia may represent a prelude to the development of cachexia. Many of the pathophysiological mechanisms underlying sarcopenia overlap with processes favoring the development of cachexia, suggesting the existence of a “wasting continuum” that leads to the progression from sarcopenia to cachexia in patients with heart failure [19]. Cachexia is a condition distinct from sarcopenia, as it is characterized by loss of total body mass and multiple tissues, including muscle, adipose tissue, and bone. Cachexia primarily affects patients with advanced CHF, typically those in higher New York Heart Association (NYHA) functional classes [20]. It is also associated with significantly higher mortality among patients with CHF. Anker et al. demonstrated a 50% mortality rate among patients with CHF and cachexia over 18 months, identifying cachexia as a strong negative prognostic factor for mortality in CHF [21].

Comparative studies have demonstrated that the presence of a sarcopenic component is associated with the lowest levels of muscle mass and muscle strength, as reflected by reduced handgrip strength, quadriceps muscle strength, six-minute walk distance (6MWT), peak oxygen uptake (pVO_2_), and Short Physical Performance Battery (SPPB) scores. Patients with skeletal muscle loss exhibited a lower quality of life (QOL) compared with cohorts with cachexia or without either of these comorbid conditions. Sarcopenia in patients with chronic heart failure may represent a key process increasing hospitalization rates, limiting independence and quality of life, and—through an increased predisposition to the development of cachexia—raising the risk of mortality [13].

## 6. Pathophysiology of Sarcopenia in Chronic Heart Failure

Cruz-Jentoft et al. highlighted aging, low physical activity, malnutrition, and systemic illness as key factors that may predispose individuals to sarcopenia. When factors other than advanced age are present, sarcopenia is referred to as secondary sarcopenia, as it is induced by an underlying pathology [5].

The frequent coexistence of secondary sarcopenia in patients with chronic heart failure can be attributed to multiple disturbances predisposing to its development, resulting from the impact of heart failure on the body and skeletal muscle tissue, as well as from concomitant malnutrition and reduced physical activity.

### 6.1. Alterations in Skeletal Muscle Tissue

Numerous histopathological alterations characteristic of secondary sarcopenia have been observed in the skeletal muscle of patients with chronic heart failure. These include atrophy of type I (slow-twitch oxidative) and type IIA (fast-twitch oxidative) fibers, accompanied by an increased proportion of type IIB (glycolytic) fibers [22,23]. Alterations in the skeletal muscle capillary network have also been observed, including reduced capillary density and a decreased capillary-to-fiber ratio [24]. In addition, multiple mitochondrial abnormalities have been demonstrated in skeletal muscle myocytes, indicative of impaired oxidative metabolism, such as reduced volumetric and surface density of mitochondrial cristae and decreased cytochrome c oxidase activity [23]. Comparisons between myocytes from patients with chronic heart failure and age-matched healthy individuals revealed a higher number of apoptotic nuclei in patients with heart failure [25].

The morphology of sarcopenic changes in the course of chronic heart failure differs from that observed in primary sarcopenia. In contrast to heart failure-related changes, primary sarcopenia is characterized predominantly by atrophy of type II fibers, preservation of type I fibers, and infiltration of muscle tissue by adipocytes. These changes are largely dependent on motor unit denervation, rather than on the alterations caused by reduced cardiac output in chronic heart failure [26].

### 6.2. Main Factors in the Pathophysiology of Sarcopenia in CHF

Among the most important factors inducing sarcopenia in heart failure are hormonal and neurohormonal disturbances, chronic oxidative stress with subsequent apoptosis, as well as endothelial dysfunction and hemodynamic abnormalities within skeletal muscle tissue.

#### 6.2.1. Hormonal Dysregulation

Cittadini et al. demonstrated the presence of metabolic failure syndrome in 77.5% of patients with chronic heart failure and further identified this syndrome as being associated with increased mortality and more frequent hospitalizations. Deficiency of anabolic hormones, such as growth hormone, insulin-like growth factor 1 (IGF-1), and testosterone, is characteristic of metabolic failure syndrome [27]. Chronic heart failure is associated with reduced local insulin-like growth factor-1 (IGF-1) expression and impaired tissue responsiveness to growth hormone. Dysregulation of the growth hormone/IGF-1 axis disrupts the balance between cellular processes regulating hypertrophy and atrophy [28]. In the context of chronic heart failure, IGF-1 deficiency significantly contributes to reduced skeletal muscle strength and mass, leading to sarcopenia [29].

Testosterone levels decline with age; however, in patients with chronic heart failure, due to the presence of metabolic failure syndrome, testosterone concentrations decrease earlier and are lower than in an age-matched healthy population [30]. Testosterone exerts beneficial effects on skeletal muscle by promoting protein synthesis over degradation and stimulating the formation of new myotubules with an increased number of myonuclei, leading to increased muscle mass, strength, and exercise capacity [31]. Reduced testosterone levels in patients with chronic heart failure may therefore contribute to the development of sarcopenia.

#### 6.2.2. Neurohormonal Dysfunction

In addition to hormonal disturbances, chronic heart failure is characterized by excessive adrenergic stimulation, which represents one of the fundamental neurohormonal abnormalities [3]. Da Fonseca et al. demonstrated dysfunction of the sympathetic–parasympathetic axis in patients with heart failure and sarcopenia by assessing muscle sympathetic nerve activity using peroneal nerve microneurography. In the sarcopenic group, adrenergic activity was higher, reaching 47 (41–52) bursts/min, compared with patients with heart failure without sarcopenia, in whom an average of 40 (34–48) bursts/min was observed [32]. These findings indicate a negative role of excessive sympathetic stimulation in the development of sarcopenia in chronic heart failure.

#### 6.2.3. Increased Myostatin Expression

Elevated levels of myostatin (MSTN), a hormone that negatively regulates myocyte cellular processes, have also been observed in chronic heart failure [33]. Animal models have demonstrated a detrimental effect of increased myostatin expression by cardiomyocytes on skeletal muscle atrophy [34]. The myostatin pathway is activated only during severe cardiac stress and limits uncontrolled organ growth. Reducing skeletal muscle mass, it decreases the circulatory load imposed on the heart. However, in the long term, chronic myostatin stimulation induces sarcopenia, markedly limiting patients’ exercise capacity [35].

#### 6.2.4. Chronic Inflammation

Both heart failure with preserved ejection fraction and reduced ejection fraction are associated with elevated systemic inflammatory markers, such as tumor necrosis factor alpha (TNF-α), interleukins 1 and 6, and galectin-3. It is currently unclear how pro-inflammatory cytokines influence cardiac remodeling and whether they represent a cause or a consequence of disease progression [36]. Schaap et al. demonstrated a negative impact of elevated pro-inflammatory cytokine levels on muscle strength and mass, identifying chronic inflammation as one of the probable causes of sarcopenia [37]. Elevated pro-inflammatory cytokines increase the expression of Murf-1 mRNA and protein, which upregulates the ubiquitin–proteasome system in myocytes [38].

#### 6.2.5. Oxidative Stress and Reactive Oxygen Species (ROS)

Another factor likely to exacerbate sarcopenic processes is oxidative stress and increased production of reactive oxygen species [39]. In addition to an acute increase in oxidative stress mediators, heart failure is also characterized by depletion of endogenous antioxidant defense mechanisms, which may contribute to skeletal muscle loss [40]. Excessive amounts of ROS can trigger mitochondrial degradation and damage the DNA of myonuclei. Additionally, oxidative stress negatively affects myogenesis [41]. Initially, oxidative stress appears to downregulate myogenic differentiation, but high concentrations of ROS may contribute to the loss of myoblast function and increased myoblast cell death [42]. High levels of ROS result in reduced p21 promoter activity, which exacerbates the apoptosis of myoblasts [43]. Elevated levels of ROS also compromise muscle contractile function by decreasing myofibrillar Ca^2+^ sensitivity, which may lead to a reduction in the resistance of skeletal muscles to fatigue [44].

Oxidative stress may impair muscle tissue regeneration by affecting skeletal muscle satellite cell function [41]. At the tissue level, accumulation of ROS induces adipogenic differentiation of skeletal myofiber-associated cells [45]. Overall, oxidative stress accelerates sarcopenic muscle loss, contributing to reduced physical performance.

#### 6.2.6. Disturbed Hemodynamics and Endothelial Dysfunction

Hemodynamic disturbances resulting from reduced cardiac output also contribute to skeletal muscle wasting in patients with chronic heart failure. Endothelial dysfunction, caused by impairment of the nitric oxide–cGMP signaling pathway, results in reduced vasodilation and consequently altered peripheral blood flow. This pathology is systemic and may persist even after heart transplantation [46]. An abnormal hyperemic response in the vasculature results in reduced blood flow to skeletal muscles, leading to decreased muscle strength and physical performance [47]. In healthy individuals, activation of the skeletal muscle metaboreflex during exercise increases vascular resistance as well as cardiac contractility and stroke volume. In contrast, patients with heart failure exhibit an altered response: reduced cardiac output leads to skeletal muscle ischemia, which in turn increases catabolism and structural changes. This ultimately limits exercise capacity and leads to excessive activation of the metaboreflex [16]. Heart transplantation does not eliminate the excessive activation of the metaboreflex either, which is one of the reasons why exercise capacity in heart transplant recipients remains lower compared with healthy cohorts [48].

#### 6.2.7. Malnutrition and Anorexia

Sze et al., using nutritional status indices such as the Geriatric Nutritional Risk Index (GNRI) and the Controlling Nutritional Status (CONUT) score, demonstrated that malnutrition is a common abnormality among ambulatory patients with heart failure and that it is strongly correlated with increased mortality [49].

Anorexia, a frequent symptom of heart failure, is independently associated with reduced muscle mass and muscle strength. Moreover, patients with sarcopenia have been shown to have decreased intake of several micronutrients, including iron, phosphorus, magnesium, potassium, and vitamin K. The causes of anorexia in patients with chronic heart failure are mostly attributed to pulmonary and gastrointestinal congestion, increased resting energy catabolism, and certain pharmacological agents used in heart failure therapy, such as digoxin [50,51,52,53,54].

**Figure 1:**
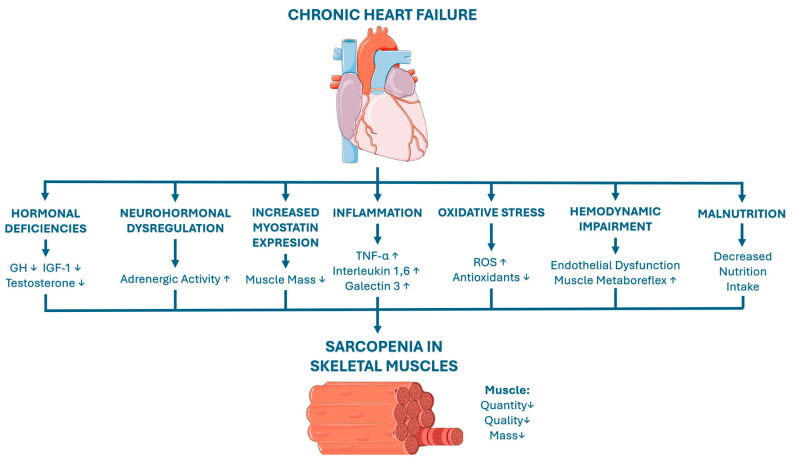
Pathophysiological pathways of CHF-induced sarcopenia in skeletal muscles. Arrows indicate direction of change: ↑ increase, ↓ decrease. Heart icon and muscle-fiber icon from Servier Medical Art (https://smart.servier.com/), licensed under CC BY 3.0 (https://creativecommons.org/licenses/by/3.0/).

A graphical summary is presented in Figure 1.

### 6.3. Pathophysiological Differences in Sarcopenia Between HFrEF and HFpEF

The mechanisms and clinical expression of sarcopenia differ substantially between HFrEF (heart failure with reduced ejection fraction) and HFpEF (heart failure with preserved ejection fraction). Available data suggest a prevalence of approximately 30–40% in HFrEF and 25–30% in HFpEF, with variability across studied populations [7].

In HFrEF, skeletal muscle dysfunction is primarily driven by reduced cardiac output, neurohormonal activation, and catabolic signaling, leading to mitochondrial impairment and reduced oxidative capacity [22,23,25]. In contrast, HFpEF is characterized by a systemic metabolic phenotype associated with aging, obesity, and chronic inflammation [55].

Patients with HFpEF frequently present with sarcopenic obesity, where increased adiposity coexists with impaired skeletal muscle function. In this setting, altered adipokine signaling and microvascular dysfunction contribute to impaired skeletal muscle perfusion and reduced oxygen utilization, resulting in exercise intolerance that may be disproportionate to central cardiac impairment [47,56].

These findings support the need for phenotype-specific approaches to the assessment and management of sarcopenia in heart failure.

### 6.4. Body Composition and Hydration Status in Heart Failure

Accurate assessment of skeletal muscle mass in heart failure is challenging due to fluid overload and altered body water distribution. Clinically apparent congestion occurs relatively late, meaning that subclinical fluid accumulation may already affect body composition measurements.

Bioelectrical impedance analysis (BIA), widely used in clinical practice, assumes normal body water distribution. In heart failure, expansion of the extracellular compartment reduces electrical resistance and may lead to overestimation of lean body mass, potentially masking sarcopenia [57].

Methods such as bioelectrical impedance spectroscopy allow differentiation between intracellular and extracellular water, while imaging techniques such as dual-energy X-ray absorptiometry or computed tomography may improve accuracy [58]. However, all methods should be interpreted with caution in the presence of fluid overload.

For this reason, body composition assessment should be performed under euvolemic conditions whenever possible and interpreted in the clinical context.

## 7. Nutritional and Pharmacological Interventions Targeting Sarcopenia in Chronic Heart Failure

### 7.1. Nutritional Interventions

Therapeutic options for sarcopenia in chronic heart failure remain limited. Physical exercise and nutritional interventions, particularly high-protein diets, represent the cornerstone of prevention and treatment. Ongoing research is focused on identifying effective pharmacological strategies aimed at attenuating the pathological loss of skeletal muscle mass and function in patients with heart failure.

The primary objective of dietary intervention is to restore the balance between anabolic and catabolic processes within skeletal muscle tissue. Increased daily protein intake has been proposed as a strategy to counteract sarcopenia [59]. Aquilani et al. demonstrated that adequate intake of protein- and amino acid-rich meals, adjusted to individual energy requirements, improved exercise capacity, including peak oxygen uptake and walking performance, in stable patients with chronic heart failure and concomitant sarcopenia; however, no significant improvement in skeletal muscle metabolic abnormalities was observed [60].

Rozentryt et al. reported that high-calorie, energy-dense nutritional supplementation resulted in increased fat and muscle mass and improved quality of life. A concomitant reduction in circulating tumor necrosis factor-α levels was observed, suggesting a potential anti-inflammatory effect [61].

Additionally, supplementation with branched-chain amino acids, particularly leucine, may further stimulate skeletal muscle protein synthesis [62].

Although no large studies have evaluated micronutrient supplementation in patients with both chronic heart failure and sarcopenia, available evidence indicates that vitamin D administration in individuals with reduced serum levels may improve muscle strength [15]. 

Iron deficiency is common among patients with chronic and acute heart failure. Suitable supplementation may improve exercise capacity and reduce the risk of hospitalization, as demonstrated by Ponikowski et al. [63].

It should be emphasized that most studies evaluating dietary interventions for sarcopenia have been conducted in elderly populations without significant comorbidities. High-protein diets should therefore be applied cautiously to patients with chronic heart failure and coexisting conditions, such as chronic kidney disease or diabetes mellitus [64,65].

### 7.2. Pharmacological Interventions

Potential pharmacological approaches to sarcopenia are largely based on the pleiotropic effects of drug classes routinely used in the treatment of chronic heart failure, including angiotensin-converting enzyme inhibitors (ACEIs), β-adrenergic receptor blockers, and mineralocorticoid receptor antagonists (MRAs).

ACEIs, which constitute first-line therapy for heart failure, have been suggested to exert beneficial effects on skeletal muscle [1]. Ata et al. reported a lower prevalence of sarcopenia among hypertensive patients treated with ACEIs compared with those receiving angiotensin II receptor blockers or other antihypertensive agents [66]. In addition, ACEIs may exert anti-inflammatory effects and increase insulin-like growth factor-1 levels in skeletal muscle [67]. However, data regarding their direct impact on muscle function remain inconsistent, warranting further investigation [68,69].

Beyond their central hemodynamic effects, MRAs may exert favorable peripheral actions relevant to sarcopenia. Spironolactone has been shown to improve endothelial function in skeletal muscle vasculature, enhance nitric oxide bioavailability, and inhibit local angiotensin II formation, potentially improving tissue perfusion and exercise capacity [70,71]. MRAs may also reduce the production of pro-inflammatory cytokines implicated in sarcopenia and favorably influence skeletal muscle metabolism and apoptosis [70,72].

β-Adrenergic receptor blockers represent another drug class with potential effects on skeletal muscle. Espindolol has been shown to increase muscle mass and handgrip strength in selected populations [73]. Additionally, animal models have demonstrated that carvedilol increases skeletal muscle fiber contractility, which may translate into enhanced skeletal muscle strength. No hypertrophic effects in myocytes were shown [74].

Given the frequent hormonal disturbances observed in sarcopenia and heart failure, several investigational therapies targeting endocrine pathways have been explored. These include testosterone supplementation, selective androgen receptor modulators (SARMs), ghrelin-based therapies, and myostatin inhibitors [75,76].

Testosterone deficiency, commonly observed in heart failure, is associated with reduced physical capacity [19]. Testosterone replacement therapy, administered at physiological doses, has been shown to improve exercise capacity, peak oxygen uptake, and muscle strength, despite inconsistent effects on muscle mass [77,78,79]. However, potential adverse effects, including prostate hypertrophy and increased thromboembolic risk, limit its routine clinical use [75].

SARMs are not currently approved for routine clinical use. These agents stimulate anabolic processes while minimizing androgenic adverse effects [79]. Clinical studies have demonstrated increases in body mass and, in some cases, muscle strength; however, results remain inconsistent, and data in heart failure populations are limited [80,81]. Further investigation is required to establish their safety and efficacy in this setting [76].

Ghrelin levels are often reduced in elderly patients with sarcopenia. In a small clinical study, intravenous administration of synthetic ghrelin improved left ventricular ejection fraction, reduced circulating norepinephrine levels, and enhanced exercise capacity and skeletal muscle mass in patients with heart failure [82]. Larger randomized trials are needed to confirm these findings.

Inhibition of myostatin represents another potential therapeutic strategy. Although experimental models have demonstrated anabolic effects, clinical trials have yielded mixed results [83,84]. A lack of specificity in many of the myostatin inhibitors could account for unsatisfactory clinical trials. Myostatin shares many structural similarities with other factors from the TGF-β superfamily, mainly GDF11, which leads to cross-reaction effects or reduced efficacy [85]. One of the most extensively studied MSTN inhibitors is bimagrumab. Initially, studies showed promise, with increases in lean muscle mass and 6MWT, but more extensive trials found no significant improvements in 6MWT and overall mobility [86]. Apitegromab is the first of this class to seek FDA approval, but only in spinal muscular atrophy [87]. The focus of the research shifted mainly to their use in obesity, both alone and in combination with glucagon-like peptide-1 receptor agonists (GLP-1 RAs) [88].

Yamakage et al. reported a reduction in MSTN levels after administering dapagliflozin but without an increase in muscle mass [89].

Sarcopenic obesity represents a distinct phenotype characterized by the coexistence of skeletal muscle loss and increased adiposity. This phenotype has been reported in approximately 19.4% of patients with chronic heart failure [90]. Weight reduction therapy by GLP-1 receptor agonists, while beneficial for lowering fat mass, may negatively impact skeletal muscle mass [91]. Emerging combination therapies involving GLP-1 RAs and MSTN inhibitors aim to preserve muscle mass during weight-reducing treatment; however, dedicated studies in heart failure patient cohorts are required to evaluate their safety and efficacy [88,92].

A summary comparing the available treatment methods for sarcopenia in chronic heart failure is presented in Table 1.

## 8. Physical Training

Physical exercise is currently regarded as the most effective strategy for the prevention and treatment of sarcopenia [76]. It represents a safe, low-cost, and widely accessible intervention that, when appropriately tailored to the patient’s individual capabilities, can significantly improve exercise capacity and quality of life [93,94,95]. The most commonly applied modalities include aerobic training, resistance training, or combined exercise programs incorporating both forms of activity [93]. Studies evaluating the effects of various training modalities in patients with heart failure have demonstrated numerous beneficial adaptations in both skeletal muscle tissue and circulating biochemical parameters [76].

Aerobic training includes activities such as walking, treadmill exercise, and stationary cycling. Studies assessing the impact of aerobic exercise on health outcomes and quality of life in patients with heart failure have demonstrated increases in pVO_2_ and IGF-1 levels. Concomitantly, reductions in NT-proBNP, myostatin, TNF-α, and the expression of TRIM63 mRNA and protein have been observed [93,95].

Gielen et al. demonstrated a significant reduction in MuRF-1 expression after only four weeks of aerobic training (four sessions per week, 20 min per session) in patients with heart failure [38].

Lenk et al. reported a 36% decrease in myostatin mRNA expression and a 23% reduction in circulating myostatin protein levels in peripheral skeletal muscles after 12 weeks of regular exercise training, compared with a sedentary control group [96].

Lelyavina et al. assessed clinical and exercise-related parameters in patients with (NYHA) class III heart failure before and after 12 weeks of walking performed four to five times per week. Aerobic training resulted in increased pVO_2_, left ventricular ejection fraction (LVEF), exercise tolerance, and quality of life in approximately 30% of patients, while 95% of participants exhibited improvement in at least one of the evaluated parameters [97]. Moreover, aerobic exercise has been associated with improved survival and a reduced rate of hospitalization in patients with chronic heart failure [98].

A study conducted by Williams et al. demonstrated that circuit-based resistance training in patients with chronic heart failure led to increased activity of oxidative enzymes and markers, including mitochondrial maximally activated ATP production rate, β-hydroxyacyl coenzyme A dehydrogenase, enhanced citrate synthase activity, and an elevated lactate threshold—parameters strongly associated with improvements in pVO_2_. An increase in the capillary-to-muscle fiber ratio was also observed [99].

Pu et al. investigated the effects of high-intensity resistance training performed three times per week for 10 weeks, including leg press, chest press, knee extension, knee flexion, and triceps exercises. The intervention resulted in increased muscle strength and endurance as well as a greater six-minute walk distance. Skeletal muscle oxidative capacity improved significantly; however, no significant changes were observed in mean muscle fiber cross-sectional area or total body muscle mass. Peak VO_2_ remained unchanged [100].

Additional studies have evaluated the effects of different exercise modalities in older adults with sarcopenia by assessing knee extensor strength, gait speed, chair-stand performance, and the timed up-and-go test as measures of muscle strength and physical function. Comparative analyses indicate that the choice of training modality is associated with selective improvements in specific functional outcomes. Resistance and combined training exerted favorable effects on gait speed and knee extensor strength, whereas whole-body vibration training did not produce significant improvements in these parameters. All three modalities—resistance, combined, and whole-body vibration training—were associated with improved timed up-and-go performance but did not significantly affect chair-stand test results [101].

## 9. Conclusions and Further Directions

Sarcopenia is a well-established prognostic factor associated with adverse clinical outcomes in patients with heart failure and contributes to increased morbidity and mortality. Since the pathophysiological changes leading to skeletal muscle loss in patients with heart failure involve multiple mechanisms and factors, it is unlikely that a single intervention will be sufficient. Therefore, a multimodal intervention that combines exercise, nutrition, and pharmacotherapy will be needed to address the various pathways that contribute to skeletal muscle loss in patients with heart failure [7].

Several pharmacologic agents, such as myostatin inhibitors, ghrelin-based therapies, and selective androgen receptor modulators, are currently being investigated to target skeletal muscle metabolism. Although these agents show promise in preclinical studies, limited clinical data are available to support their use in the management of skeletal muscle atrophy in heart failure patients [82,83,84,89].

Therefore, several clinical trials are currently underway to evaluate the impact of multimodal lifestyle-based interventions that combine structured physical activity with tailored dietary strategies. These studies will assess whether higher protein intake combined with a structured exercise program can positively affect skeletal muscle mass, strength, and functional ability in patients with heart failure [15].

The PROT-HF study (NCT03142399) is designed to evaluate the efficacy of whey protein supplementation combined with a structured exercise program on muscle mass, muscle strength, and quality of life in patients with chronic heart failure [102]. An additional mechanistically oriented study (NCT05627440) is evaluating the effectiveness of a skeletal muscle recovery protocol consisting of tailored nutritional supplements and structured physical activity in patients with HFrEF [103]. Most recently, the randomized, double-blind STRIVE trial (NCT06428192) was initiated to evaluate the additive effects of whey protein supplementation and progressive resistance training in patients with HFpEF [104].

Taken together, current data demonstrate that skeletal muscle dysfunction is a crucial extracardiac component of the heart failure syndrome and significantly impacts both the rate of functional decline and long-term survival in patients with HF. Effective management of sarcopenia will likely require integrated strategies combining optimized heart failure therapy with targeted nutritional interventions and structured exercise programs [15]. Future research should focus on well-designed randomized trials to determine whether multimodal approaches aimed at improving skeletal muscle metabolism can translate into meaningful improvements in functional capacity, quality of life, and long-term clinical outcomes in patients with heart failure [105].

## Figures and Tables

**Table 1:** Therapeutic strategies targeting sarcopenia and cachexia in patients with chronic heart failure.

Therapeutic Approach	Targeted Mechanism	Evidence and Clinical Impact	Limitations and Considerations
Resistance and aerobic exercise training	Stimulation of muscle hypertrophy; enhancement of mitochondrial function and skeletal muscle perfusion	Improves muscle strength, peak oxygen uptake (pVO_2_), and quality of life	Limited feasibility in patients with advanced heart failure or severe frailty
Nutritional support (adequate protein and caloric intake)	Correction of malnutrition; promotion of muscle protein synthesis	Associated with improvements in muscle mass and functional outcomes	Evidence remains heterogeneous; individualized nutritional assessment is required
Micronutrient supplementation (e.g., iron, vitamin D)	Correction of specific deficiencies affecting skeletal muscle metabolism	Iron repletion improves exercise capacity; vitamin D supplementation may support muscle function	Clinical benefit depends on the presence and severity of baseline deficiency
Anabolic hormone–based therapies (testosterone, growth hormone, insulin-like growth factor 1)	Restoration of anabolic–catabolic balance; stimulation of muscle protein synthesis	May improve muscle mass, strength, and exercise capacity in carefully selected patients	Safety concerns; limited efficacy in unselected populations; lack of robust outcome data
Anti-inflammatory strategies	Reduction of cytokine-mediated catabolic processes	Potential to attenuate muscle wasting	Clinical trials have not demonstrated consistent clinical benefit
Myostatin inhibition	Suppression of negative regulation of skeletal muscle growth	Demonstrates favorable effects on muscle mass in experimental models	Limited clinical evidence; long-term safety remains uncertain
Appetite stimulation and management of anorexia	Enhancement of caloric intake	May support nutritional status and preservation of muscle mass	Limited heart failure–specific evidence
Multidisciplinary rehabilitation programs	Integrated management of physical, nutritional, and psychosocial factors	Improves functional capacity and quality of life	Resource-intensive; availability varies across healthcare settings

## Data Availability

No new data were created or analyzed in this study. Data sharing is not applicable to this article.

## Abbreviations

The following abbreviations are used in this manuscript:

HF	Heart Failure
CHF	Chronic Heart Failure
NYHA	New York Heart Association
6MWT	Six-Minute Walk Test
pVO_2_	Peak Oxygen Uptake
SPPB	Short Physical Performance Battery
EQ-5D	EuroQol-5 Dimension
CPET	Cardiopulmonary Exercise Testing
VE/VCO_2_ slope	Ventilatory Equivalent for Carbon Dioxide Slope
IGF-1	Insulin-Like Growth Factor 1
TNF-α	Tumor Necrosis Factor Alpha
ROS	Reactive Oxygen Species
mtDNA	Mitochondrial Deoxyribonucleic Acid
GNRI	Geriatric Nutritional Risk Index
CONUT	Controlling Nutritional Status Score
BIA	Bioelectrical Impedance Analysis
ACEI	Angiotensin-Converting Enzyme Inhibitor
MRA	Mineralocorticoid Receptor Antagonists
SARM	Selective Androgen Receptor Modulators
TRIM63	Tripartite Motif-Containing 63
MuRF-1	Muscle RING Finger 1
NT-proBNP	N-Terminal Prohormone of Brain Natriuretic Peptide
LVEF	Left Ventricular Ejection Fraction
ET	Exercise Tolerance
QOL	Quality of Life
HFpEF	Heart Failure Preserved Ejection Fraction
HFrEF	Heart Failure Reduced Ejection Fraction
MSTN	Myostatin
TGF-β	Transforming Growth Factor-Beta
GDF11	Growth Differentiation Factor 11
GLP-1 RA	Glucagon-like Peptide-1 Receptor Agonists
cGMP	Cyclic Guanosine Monophosphate
